# Distinguishing between Photothermal and Photoelectric Effects in Li-Ion Batteries

**DOI:** 10.1021/acselectrochem.4c00212

**Published:** 2025-02-07

**Authors:** Lifu Tan, Byung-Man Kim, Kohei Shimokawa, Su Jin Heo, Arvind Pujari, Michael De Volder

**Affiliations:** † Institute for Manufacturing, Department of Engineering, 2152 University of Cambridge, Cambridge CB3 0FS, United Kingdom; ‡ Cambridge Graphene Centre, 2152 University of Cambridge, Cambridge CB3 0FA, United Kingdom; § Frontier Research Institute for Interdisciplinary Sciences, Tohoku University, 6-3 Aramaki Aza Aoba, Aoba-ku, Sendai 980-8578, Japan; ∥ Institute for Materials Research, Tohoku University, 2-1-1 Katahira, Aoba-ku, Sendai 980-8577,Japan; ⊥ Cavendish Laboratory, Department of Physics, University of Cambridge, Cambridge CB3 0HE, United Kingdom

**Keywords:** Photothermal effect, photoelectric effect, light−matter interaction, photo-batteries, energy storage mechanisms, bandgap engineering

## Abstract

Over the past decades, photo-enhanced batteries where light is used to improve the rate performance or recharge batteries have received increased attention in the academic community. However, the underlying mechanisms that contribute to performance enhancement in several photo-enhanced batteries are still under debate. For instance, photothermal effects, resulting from light absorption and subsequent conversion to heat through non-radiative relaxation, and photo-induced charge transfer, involving the generation and separation of electron-hole pairs under the illumination resulting in charge carrier transport, can be challenging to disentangle. This study aims to distinguish between the photothermal and photo-induced charge transfer in TiO_2_ and Fe_2_O_3_ as model systems because of their photoactivity and ability to store Li-ions. Using ultraviolet photoelectron spectroscopy (UPS) and UV-vis spectroscopy, we measure the band positions of these materials, and by a combination of different electrochemical processes, we demonstrate the transition from photothermal dominated to photoelectric effects in these materials. These results further illustrate the fact that different processes take place in photo-batteries, and this work provides a workflow to investigate these complex interactions.

## Graphical abstract

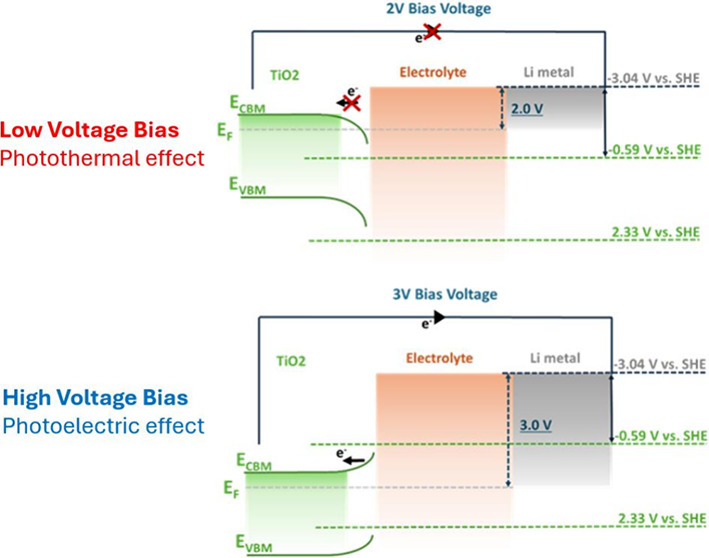

## Introduction

The development of compact energy solutions that can simultaneously operate as solar cells and batteries goes back to the 70s.^1^ With the emergence of smart off-grid sensor networks and internet of things devices, there has been a renewed interest in these so-called photo-batteries during the past decade.^2^ In particular, two-electrode systems where a photoactive cathode is simultaneously used as a Li-ion battery (LIB) cathode material and photoactive component have gained popularity, particularly because of their compact design. However, the interaction of light and battery electrodes is complicated, and in particular, the effects of heat generation as a result of exposing the electrodes to light is very difficult to eliminate.^3^ In particular, LIB electrodes are typically mixed with carbon additives to enhance the electrical conductivity of electrodes which results in high emissivity coatings that are prone to heating by radiative heat transfer.^4^ Furthermore, the active materials used in photo-batteries are often photothermally active and change bandgap with their stage of charge, which further complicates the analysis of photo-charging effects.^5,6^ Finally, the operation of these batteries are complicated by side reactions with the electrolyte and capacitive effects.^4,7^

In this paper, we investigate light interaction with TiO_2_ and Fe_2_O_3_ LIB electrodes, which are known to be photothermally active, and have been reported in literature as photo-batteries.^8,9^ We implement a combination of electrochemical impedance spectroscopy (EIS) based method to monitor the cell temperature in situ and chrono-amperometry with different voltage biases and pulsed light, to demonstrate a transition in photo-battery operation from photothermal to photo-induced charge transfer. We hope this work can contribute to advancing the understanding of the intricate mechanisms governing the interaction between light and battery performance and help better explain improvements in electrochemical performance observed in photo-batteries under illumination.

## Experimental Section

### Material Synthesis and Photoelectrode Preparation

The electrode slurry is creating by mixing anatase/rutile TiO_2_ or Fe_2_O_3_ nanoparticles, carbon black Super-P, *N*-Methyl-2-pyrrolidone (NMP), and polyvinylidene fluoride (PVDF) and drop-casting on carbon felt (Toray Carbon Paper TGP-H-060). NMP, PVDF, anatase/rutile TiO_2_, and Fe_2_O_3_ nanoparticles were purchased from Sigma-Aldrich. The carbon felt is first cut with a diameter of 10 mm and pretreated in a UV-Ozone machine. 20 μL battery slurry was drop-casted at the center of the carbon felt and the mass loading of the active material is kept at ∼1 mg/cm^2^. The coated electrodes were dried on a hot plate overnight. The weight of the electrodes was measured using a micro-balance before and after drop-casting in order to calculate the mass loading of the active material.

### Material Characterization

The morphologies of the anatase/rutile TiO_2_ or Fe_2_O_3_ nanoparticles were examined using scanning electron microscopy (Phenom-SEM). X-ray diffraction (XRD) patterns were recorded using a Bruker D8 Advance instrument with Cu *K*_α_ radiation and a scan rate of 6° min^–1^. Optical properties and the bandgap of the material were determined using a PerkinElmer UV/Vis/NIR Spectrometer (Lambda 750). UPS analysis was performed using a Thermo Scientific Escalab 250Xi fitted with a helium lamp, which produces a He I (21.2 eV) source.

### Design of the Photo-battery

To assemble the photo-enhanced lithium-ion battery (photo-LIB), a 10 mm diameter hole was drilled in a coin cell (CR2032) can, and a glass window was placed over it. The glass was sealed by using epoxy glue. The photocathode was positioned on the glass window, and aluminum strips were utilized to establish connections between the photocathode and the coin-cell casing. Following this, a piece of Whatman glass microfiber filter paper separator was placed atop the photocathode, and 70 μL of LiPF_6_ in EC/EMC (1:1) electrolyte was added. The Li metal counter electrode was then positioned on the separator. Finally, the photo-LIB was assembled by adding a spacer and spring on the Li anode side.

### Electrochemical Characterization of the Photo-LIB

The electrochemical measurements of the photo-enhanced lithium-ion batteries (photo-LIBs) were conducted using a Biologic VMP-3 galvanostat. Initially, galvanostatic discharge–charge tests (GCD) were performed under both dark and illuminated conditions (light source λ ≈ 470 nm, intensity ≈ 144 mW cm^–2^). Furthermore, AC impedance (EIS) measurements were conducted at a frequency ranging from 10 mHz to 100 kHz, with a voltage amplitude of 10 mV, in both dark and illuminated conditions. Thermal-compared chronoamperometry, EIS and GCD measurements were tested in a closed temperature-controlled incubator. In order to investigate the photothermal and photoexcitation effect, the cell was first subjected to a constant discharge current of 100 mA/g to 1.0 V vs. Li/Li^+^. Then, a constant voltage hold of 1.0 V was applied until the discharging current was higher than -5.0 μA. Lastly, a constant charging voltage of 2.0 V/3.0 V was applied to the device for 60 seconds.

## Results and Discussion

The photoelectrodes are prepared by dropcasting commercial anatase/rutile TiO_2_ and Fe_2_O_3_ on carbon felt (see Experimental Section). This carbon-based current collector is used because of its high electrical conductivity, large surface area, and mechanical stability. However, it blocks a substantial amount of light and limits the operating voltage of the battery as is can intercalate Li-ions at low voltages.^10^ Figure S1 shows the schematic diagrams of the photo-battery with the metal oxide photoelectrode and Li foil. A glass window is mounted at the top of the coin cell to enable an efficient light-enhanced performance. The optical properties of the metal oxides are tested by using ultraviolet photoelectron spectroscopy (UPS) and UV-vis spectroscopy as shown in Figure 1(a) and Figure S2. As presented in Figure 1(b), the bandgaps of these materials are estimated as ∼3.03 eV, ∼2.80 eV, and ∼2.50 eV for anatase TiO_2_, rutile TiO_2_, and Fe_2_O_3,_ respectively, by using the Tauc plot method.^11^ These results are in agreement with literature.^12–19^ Based on these UPS results, we can propose the band alignment of the metal oxides against the Li plating/stripping reaction (Li^0/+^) as calculated in Figure S3. It is worth noting that the conduction band minimums (CBMs) of the metal oxides are significantly lower than the Li plating/stripping potential, making the transportation of photo-electrons impossible. Therefore, the configurations of the metal oxide batteries do not meet physical requirements for photocharging.^6^ Figure S4­(a) shows SEM images of the anatase/rutile TiO_2_ and Fe_2_O_3_ nanoparticles. The X-ray diffraction (XRD) patterns of these nanoparticles are presented in Figure S4­(b) displaying the characteristic peaks as labelled on the figures with high crystallinity and no clear impurity phases.

**Figure 1:**
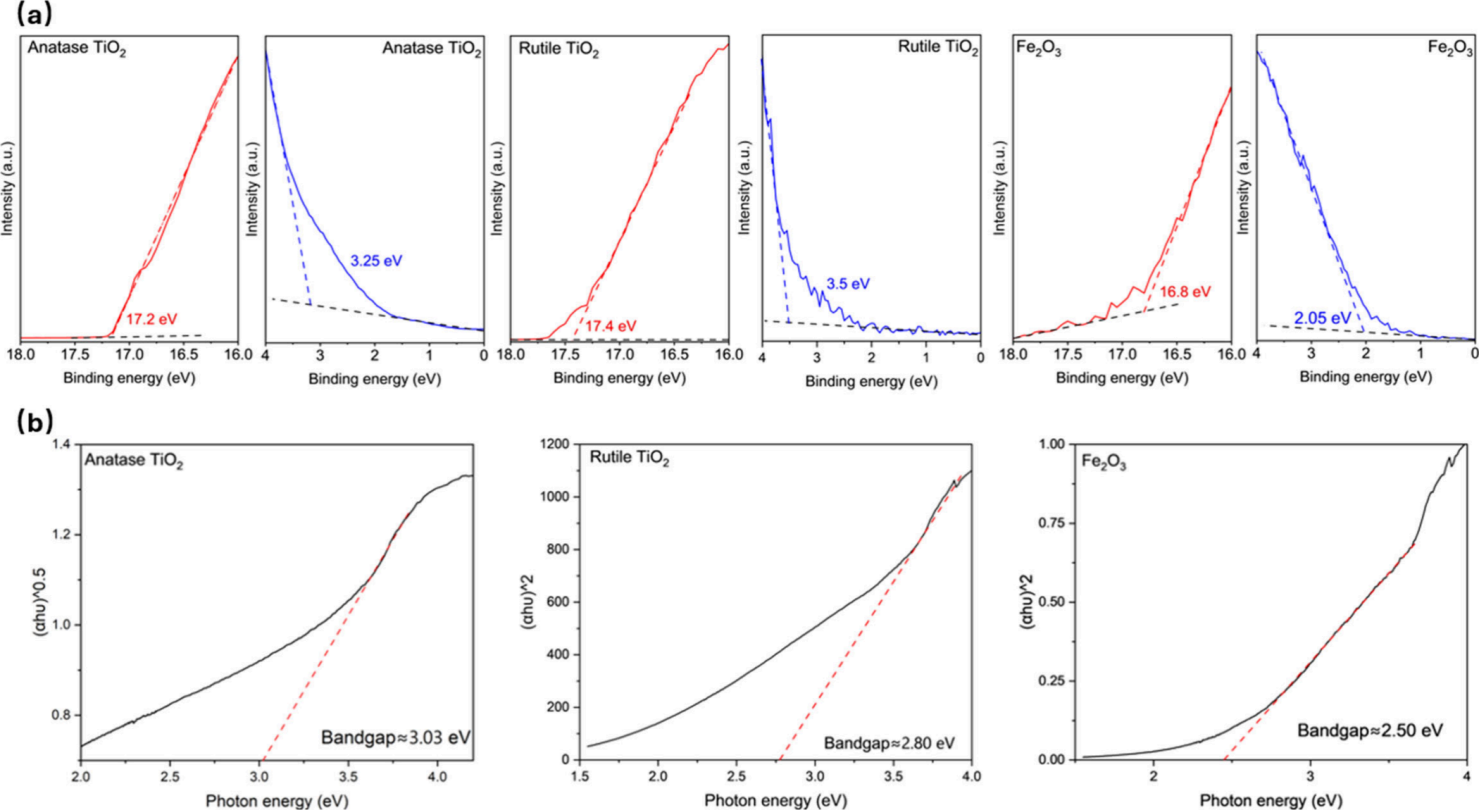
(a) UPS spectra of anatase TiO_2_, rutile TiO_2_, and Fe_2_O_3_. (b) Bandgap determination of anatase TiO_2_, rutile TiO_2_, and Fe_2_O_3_ by using the Tauc plot.

To evaluate the way exposure to light changes the internal temperature of each of the photobatteries, we used a previously studied electrochemical impedance spectroscopy (EIS) based method.^5^ For this method, the EIS response of each material is first calibrated by assembling battery cells and placing them in a temperature-controlled oven and stabilizing the temperature in the oven for 1 h before each test. Then the real part of the impedance at 100 kHz is recorded and plotted as a function of temperature, as shown in Figure S5. This calibration is then used to derive the internal temperature of the cell when exposed to light.^20^ The impedance at different temperatures is fitted using the Arrhenius-type equation as described below:

$$R\left(f_{m},T\right)=ke^{E_{A}/RT}$$

where *k* is the pre-exponential constant factor, *E*_A_ is the molar activation energy, and *R* is the gas constant. By using the fitting function, the impedances measured at various light intensities are adapted, and the relationship between light intensity and internal temperature is shown in Figure S6. We then use galvanostatic charge–discharge (GCD) tests at a current density of 100 mA g^–1^ under dark and light conditions under a blue LED at 144 mW/cm^2^ as illustrated in Figure 2(a).

**Figure 2:**
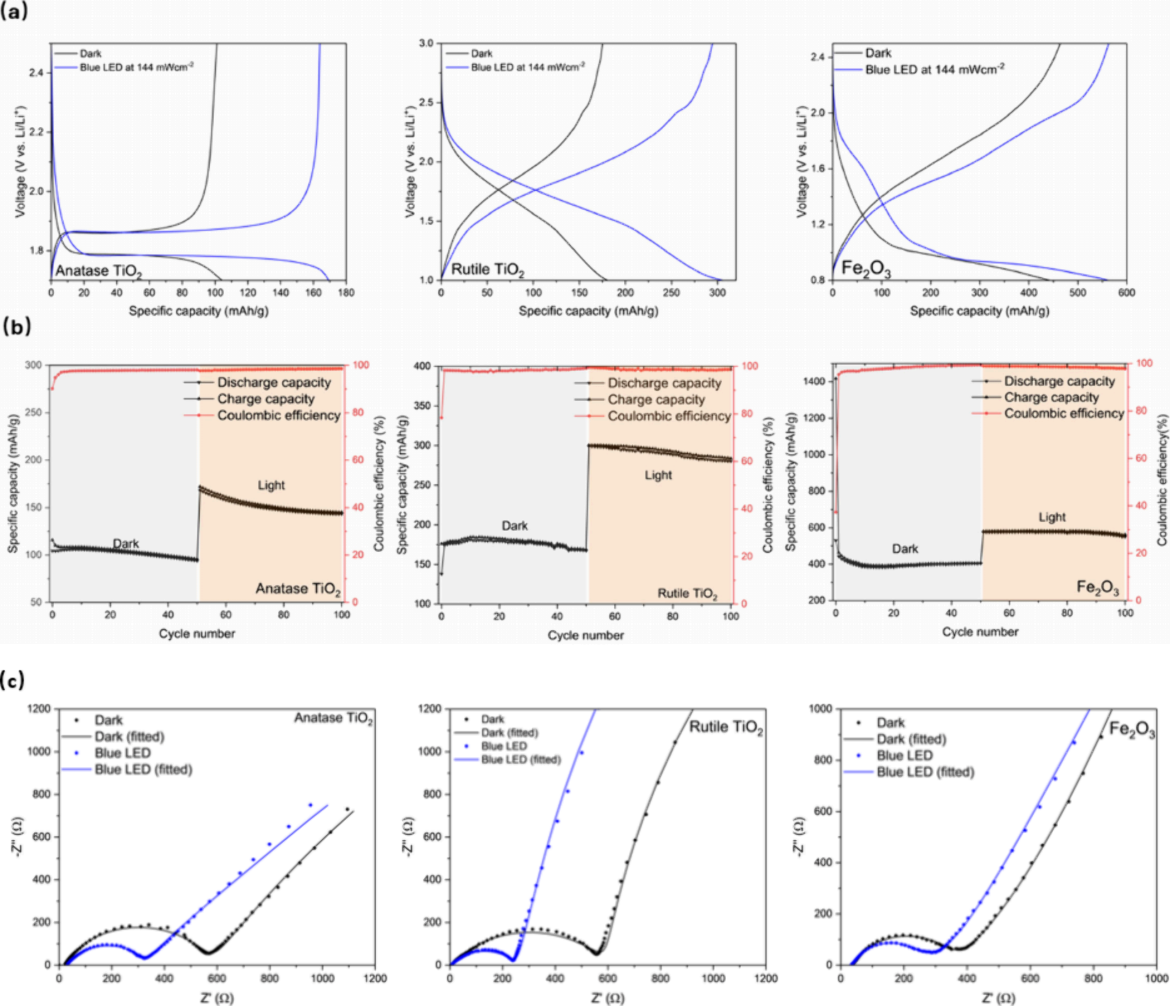
(a) Galvanostatic discharge–charge curves at 100 mA g^–1^ in dark and illuminated conditions for anatase TiO_2_, rutile TiO_2_, and Fe_2_O_3_. (b) Long-term cycling stability for anatase TiO_2_, rutile TiO_2_, and Fe_2_O_3_. (c) EIS measurement of photo-LIB obtained after the 2nd galvanostatic discharge cycle to 50% SoC in the frequency range of 10 mHz-100 kHz at 10 mV amplitude with dark and illuminated conditions for anatase TiO_2_, rutile TiO_2_, and Fe_2_O_3_.

For anatase TiO_2_, the capacity rises from 104.4 mAh g^–1^ to 169.2 mAh g^–1^, or a 62.1% increase. For rutile TiO_2_, the capacity increases from 180.8 mAh g^–1^ to 306.0 mAh g^–1^, or a 69.2% increase. The Fe_2_O_3_ shows a capacity rise of 26.0% from 443.4 mAh g^–1^ to 558.7 mAh g^–1^. We then cycle the cells for 50 cycles in the dark and 50 cycles under illuminated conditions as shown in Figure 2(b), and the anatase TiO_2_ demonstrates a capacity retention of 90.0% under dark conditions and 85.2% under illumination. Rutile TiO_2_ achieves a capacity retention of 96.7% in the dark and 93.3% under blue LED. Fe_2_O_3_ demonstrates a capacity retention of 91.2% in the dark (removing the first formation cycle) and 94.6% in light conditions. XRD measurements of the electrodes after cycling are shown in Figure S7­(a), indicating different levels of material degradation during cycling. As shown in Figure S7­(b), the difference in nominal charge and discharge voltage (Δ*V*) is lower under illumination, which indicates a lower internal impedance (see further EIS analysis). During cycling, Δ*V* increases faster for the TiO_2_ based electrodes than the Fe_2_O_3_ based ones. Under illumination, the TiO_2_ based electrodes have a slightly faster increase in over-potential, which might be due to increases in temperature or photocatalytic electrolyte decomposition.^21^ Figure 2(c) shows EIS of cells in light and dark conditions, indicating a decrease in both series and charge transfer resistance under illumination. Using the equivalent circuit for shown in Figure S8 for EIS fitting, we observe a reduction in charge transfer resistance of 50.2%, 61.0%, and 21.28% for anatase TiO_2_, rutile TiO_2_, and Fe_2_O_3_ respectively. The rate performance of the metal oxide Li-ion batteries under both dark and illuminated conditions is displayed in Figure S9, which again indicates a smaller improvement in rate performance in Fe_2_O_3_ than in the two TiO_2_ based electrodes. These trends are in-line with the lower increase in temperature increase observed in the Fe_2_O_3_ based electrodes, although other effects could be at play too.

From the measurements above, it is clear that the temperature of cells increases upon illumination and that the impedance decreases. It is, however, unclear whether improvements in performance and only due to increases in temperature or if photo-induced charge transfer also play a role. In order to investigate the photothermal and photoexcitation effect, the cell was first subjected to a constant discharge current of 100 mA/g to 1.0 V vs. Li/Li^+^. Then, a constant voltage hold of 1.0 V was applied until the discharging current was higher than −5.0 μA. Lastly, a constant charging voltage (2.0 V/3.0 V) was applied to the device for 60 seconds. By integrating the area below each curve during the constant charging voltage period as shown in Figure S10 and dividing them by the mass loading and charging time, the charging rate is defined.^22^ As shown in Figure 3, the charging rate is plotted as a function of light intensity for three types of metal oxide Li-ion batteries. In Figure 3(a), the trend during constant voltage hold charging at 2.0 V vs Li/Li^+^ is fitted using an Arrhenius-type equation, which indicates that the increasing charging rate under various light intensities is dominated by the photothermal effect. When the constant charging voltage is 3.0 V as presented in Figure 3(b), a much more linear trend is fitted from the charging rate vs light intensity results for both anatase and rutile TiO_2_ but not for Fe_2_O_3_. The linear trend corresponds to a domination of the photo-induced charge transfer over the photo-thermal effect.^22^

**Figure 3:**
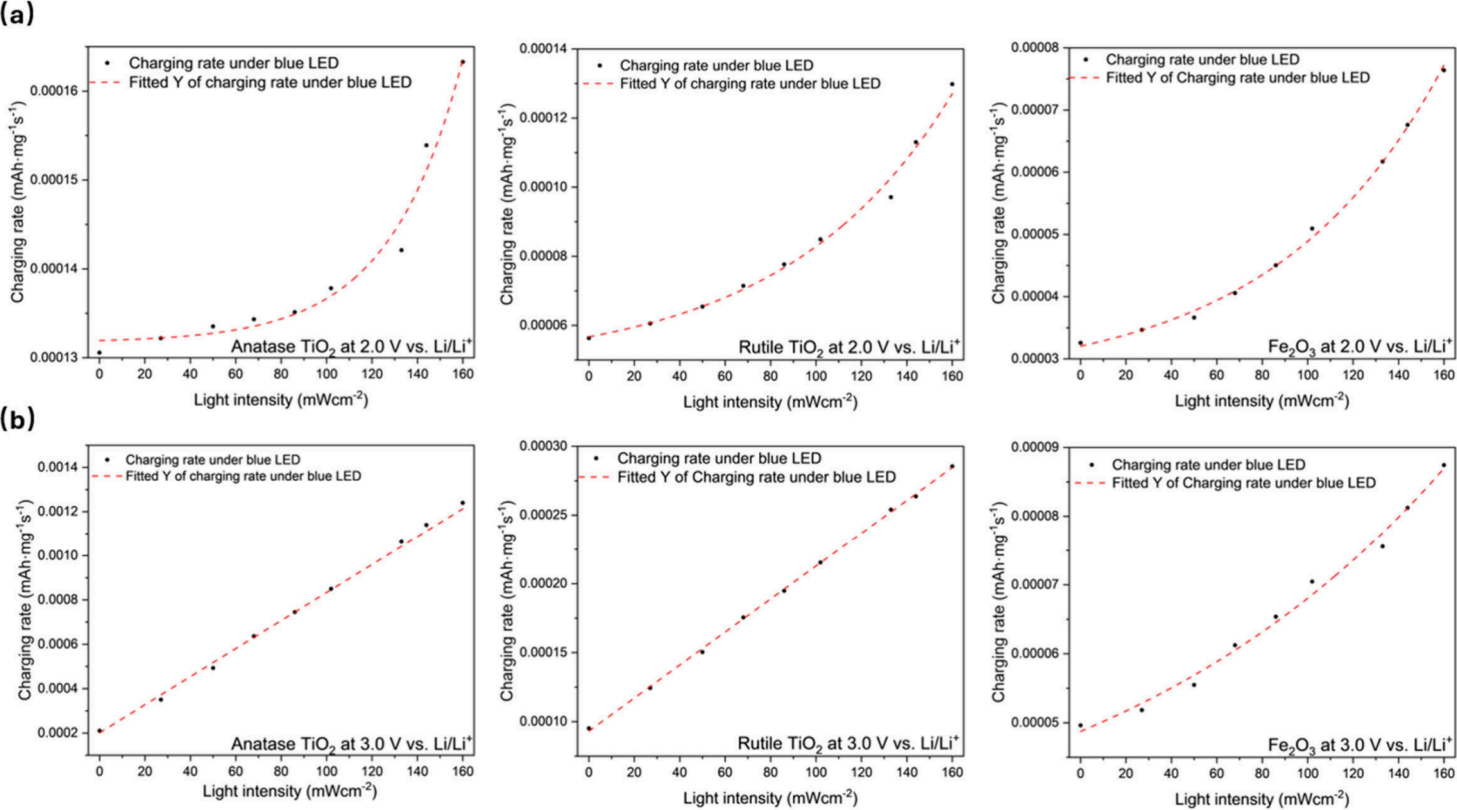
Charging rate calculated from the integration of chronoamperometry curves as a function of light intensity (blue LED) for anatase TiO_2_, rutile TiO_2_, and Fe_2_O_3_ with charging potential of (a) 2.0 V vs. Li/Li^+^ and (b) 3.0 V vs. Li/Li^+^.

To verify the observed transition from a predominantly photothermal effect to one where photo-induced charge transfer becomes significant, chronoamperometry tests were conducted. The experiments are carried out using constant voltage holds of 2.0 V vs. Li/Li^+^ and 3.0 V vs. Li/Li^+^ with intermittent light exposure (60 second on/off cycles), similar to a protocol reported recently.^8^ In their paper, band bending at the solid/solid interfaces leads to the migration of photoexcited electrons. Similar for our experiment as shown in Figure 4(a), in dark conditions, the current decays as expected for a chrono-amperometry test. However, when the light is switched on after 60 s, the current increases. At 2.0 V vs. Li/Li^+^, the current increase under illumination was minimal, due to the photothermal effect where the increase in temperature reduces cell impedance. In contrast, at 3.0 V vs Li/Li^+^ as shown in Figure 4(b), distinct behavior was observed for rutile and anatase TiO_2_, showing a rapid increase in current upon illumination. This is attributed to photo-induced charge transfer. When the light was switched off, the current responses mirrored these observations: a gradual decrease in current at 2.0 V vs. Li/Li^+^, consistent with a gradual reduction in temperature, and a sudden drop in current for rutile and anatase TiO_2_ at 3.0 V vs. Li/Li^+^. The latter indicates the cessation of photo-induced charge transfer upon removal of light. These trends corroborate the earlier findings from impedance and temperature measurements (Figure 3), further confirming the role of voltage in modulating the interplay between photothermal and photo-induced effects.

**Figure 4:**
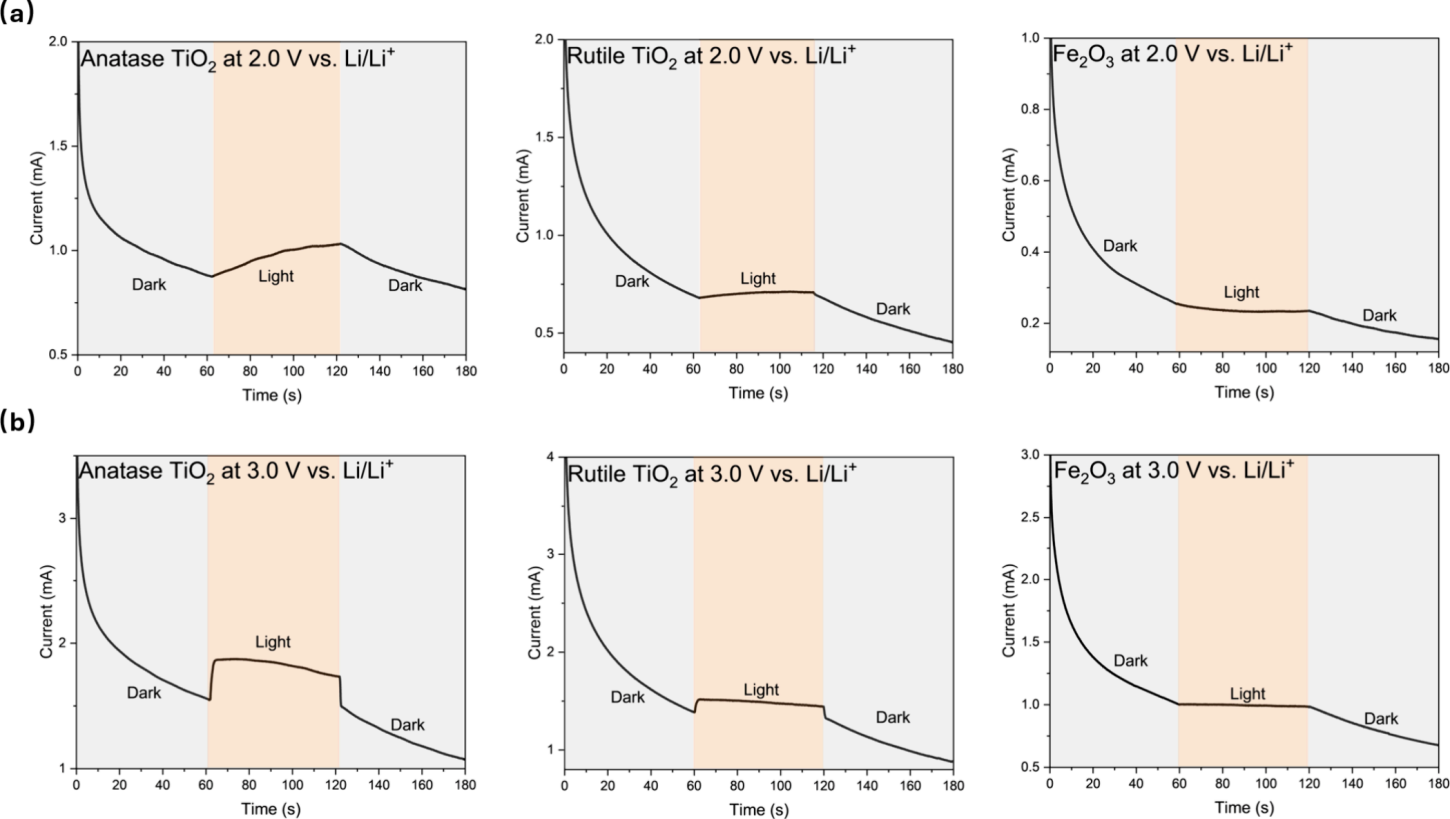
Chronoamperometry curves under intermittent light (blue LED) during constant voltage hold charging at (a) 2.0 V vs. Li/Li^+^ and (b) 3.0 V vs. Li/Li^+^.

Figure 5 illustrates the proposed energy levels in this system, with band bend similar to proposed solid electrolyte interfaces.^8^ Note that for simplicity, we are assuming that the band positions of the materials are not changing over time, whereas in reality, we know that as the CA test proceeds, the state of charge changes in a liquid electrolyte due to band bending effects at the electrode-electrolyte interface and, with it, the material band gap. In particular, in the case of conversion materials where the metal oxide is reduced to metal, the bandgap is expected to disappear altogether, which may be why the Fe_2_O_3_ electrodes only show a photothermal response. When applying 2.0 V vs Li/Li^+^ for anatase TiO_2_ as an example shown in Figure 5(a), the redox potential of Li plating (Li^0/+^) plus the bias voltage is higher than the conduction band of TiO_2_, and therefore, the photo-generated charges cannot be transferred through the external circuit. Instead, they are likely to either take part in side reactions with the electrolyte, take part in capacitive effects, or relaxation processes resulting in heating.^4,7^ On the other hand, when the bias voltage is 3.0 V, band-bending at the interface creates an internal potential that drives photo-generated electrons to the counter electrode in agreement with our observed sudden increases in photo-current.

**Figure 5:**
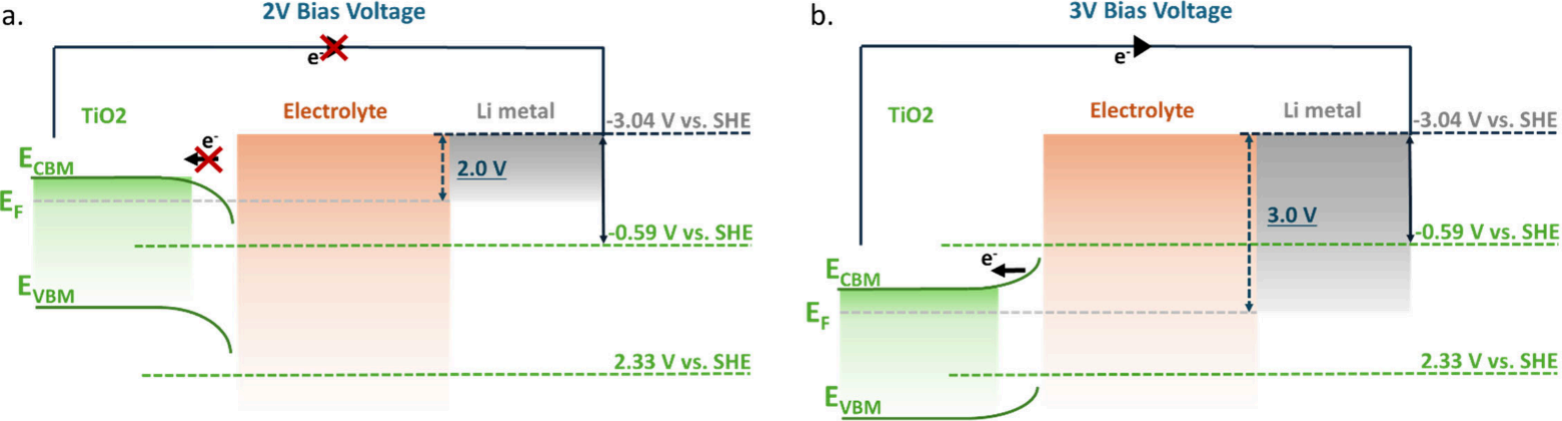
Proposed band alignment and work mechanism for photothermal and photo-induced charge transfer under (a) 2.0 V vs. Li/Li^+^ and (b) 3.0 V vs. Li/Li^+^ for anatase TiO_2_.

## Conclusions

In conclusion, we show for three different battery materials that their light response is a combination of photothermal and sometimes the photo-induced charge transfer responses. These effects can shift depending on the applied voltage bias and changes in the bandgap with state of charge. These effects can be further complicated by side reactions with the electrolyte and capacitive contributions. Overall, this work illustrates that the processes taking place in photo-batteries are intricate, and it offers new electrochemical protocols and techniques to gain insight into the mechanisms that govern the changes in behavior when illuminating photo-batteries.

## Supplementary Material



## Notes

Schematic diagrams of the photo-battery with the metal oxide photoelectrode and Li foil (Figure S1); UV-VIS spectrum of anatase TiO_2_, rutile TiO_2_, and Fe_2_O_3_ (Figure S2); Proposed band alignment against lithium plating/deplating reaction (Li^0/+^) (Figure S3); SEM imaged and XRD patterns of anatase TiO_2_, rutile TiO_2_, and Fe_2_O_3_ (Figure S4); Impedance at 100 kHz as a function of light intensities and temperature for anatase TiO_2_, rutile TiO_2_, and Fe_2_O_3_ (Fit: Arrhenius) (Figure S5); Estimated internal temperature as a function of light intensity (blue LED) TiO_2_, rutile TiO_2_, and Fe_2_O_3_. (Figure S6); (a) XRD patterns of anatase TiO_2_, rutile TiO_2_, and Fe_2_O_3_ before and after cycling and (b) the differences between nominal charge and discharge voltage (Δ*V*) as a function of cycle number under both dark and light conditions for anatase TiO_2_, rutile TiO_2_, and Fe_2_O_3_ (Figure S7); Equivalent circuit for Nyquist plots for the EIS measurement (Figure S8); Rate performance tests of the Photo-LIBs in dark and illuminated conditions for anatase TiO_2_, rutile TiO_2_, and Fe_2_O_3_ (Figure S9); Chronoamperometry curves at different light intensities (blue LED) during constant voltage hold charging (Figure S10) (PDF)
