# Supplementary material for: Distinguishing between Photothermal and Photoelectric Effects in Li-Ion Batteries
Source: ACS Electrochem. 2025 Feb 7;1(6):921–7. doi: 10.1021/acselectrochem.4c00212 (PMC12147141; doi:10.1021/acselectrochem.4c00212)
Supplement: Supplementary file 1 [file ec4c00212_si_001.pdf]

Supporting information

For

Distinguishing between Photothermal and Photoelectric Effects in Li-ion Batteries

Lifu Tan[a,b], Byung-Man Kim[a], Kohei Shimokawa[a,c,d], Su Jin Heo[a], Arvind Pujari[a,e], Michael De Volder[a,*]

[a]Institute for Manufacturing, Department of Engineering, University of Cambridge, Cambridge CB3 0FS, UK

[b]Cambridge Graphene Centre, University of Cambridge, Cambridge CB3 0FA, UK

[c]Frontier Research Institute for Interdisciplinary Sciences, Tohoku University, 6-3 Aramaki Aza Aoba, Aoba-ku, Sendai 980-8578, Japan.

[d]Institute for Materials Research, Tohoku University, 2-1-1 Katahira, Aoba-ku, Sendai 980-8577, Japan.

[e]Cavendish Laboratory, Department of Physics, University of Cambridge, Cambridge, CB3 0HE, UK

[*]Corresponding author. E-mail: mfld@cam.ac.uk

Table of Contents

UPS Calculations **S2**

Figure S1: Schematic of the photo-battery design **S2**

Figure S2: UV-Vis spectrum of anatase TiO2, rutile TiO2, and Fe2O3 .... **S2**

Figure S3: Band alignment relative to lithium plating/deplating potential **S3**

Figure S4: SEM images and XRD patterns of anatase TiO2, rutile TiO2, and Fe2O3 .... **S3**

Figure S5: Impedance as a function of light intensity and temperature **S4**

Figure S6: Estimated internal temperature as a function of light intensity **S4**

Figure S7: XRD patterns and cycling performance in dark and illuminated conditions **S5**

Figure S8: Equivalent circuit for EIS measurements **S5**

Figure S9: Rate performance tests under dark and illuminated conditions **S6**

Figure S10: Chronoamperometry curves under varying light intensities **S6**

UPS Calculations

The VBM is calculated by subtracting the sum of the He I radiation energy of 21.22 eV and the low-binding energy cut-off (LEC) from the high-binding energy cut-off (HEC). (Figure. 1a) For example, the VBM of anatase TiO_2 is calculated to be:

$$E_{VBM} = E_{HEC} - E_{He\ I} - E_{LEC}$$

$$17.2 - 21.22 - 3.25 = -7.27\ eV\ vs.\ vacuum\ level = 2.83\ V\ vs.\ SHE.$$

Considering the estimated optical bandgap of ~3.03 eV, the CBM is calculated to be:

$$E_{CBM} = E_{VBM} - E_g$$

$$2.83 - 3.03 = -0.20\ V\ vs.\ SHE.$$

Similar calculations are also done to estimate the band positions of rutile TiO_2 and Fe_2O_3.

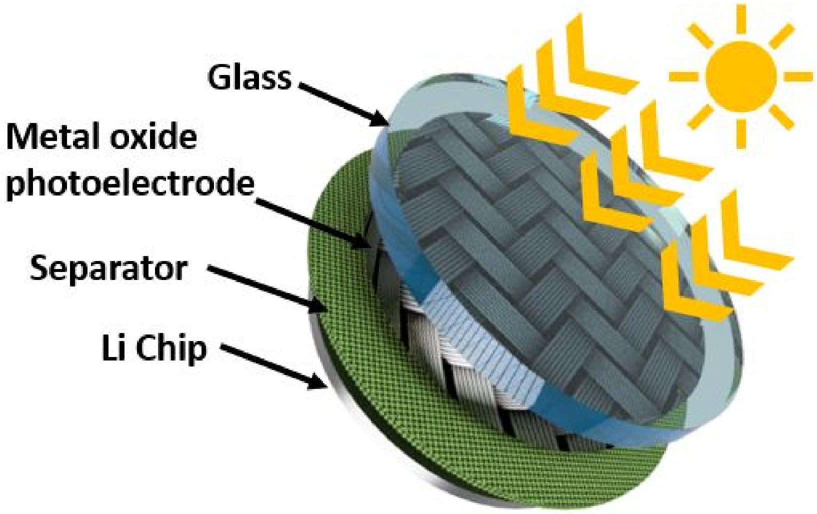

Figure S1. *Schematic diagrams of the photo-battery with the metal oxide photoelectrode and Li foil.*

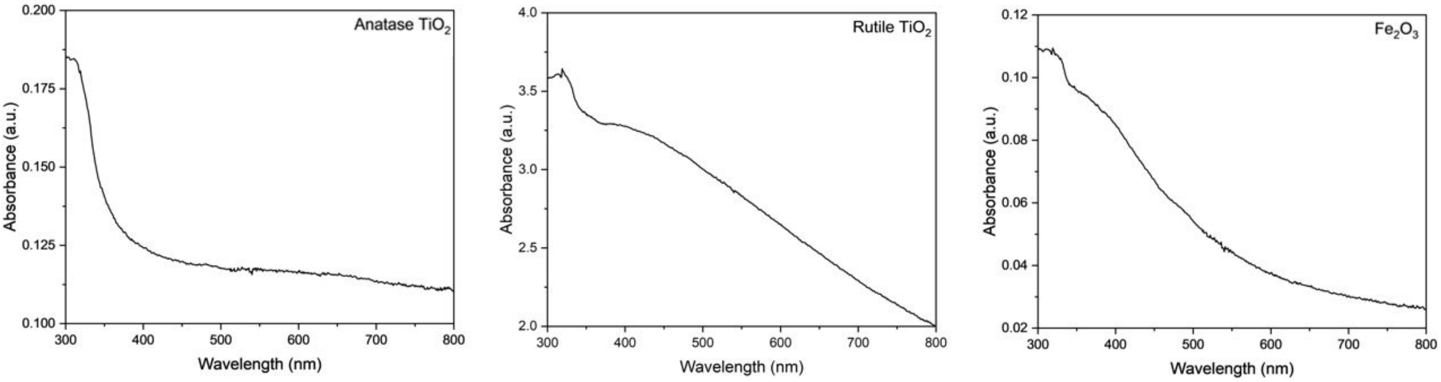

Figure S2. *UV-VIS spectrum of anatase TiO_2, rutile TiO_2, and Fe_2O_3.*

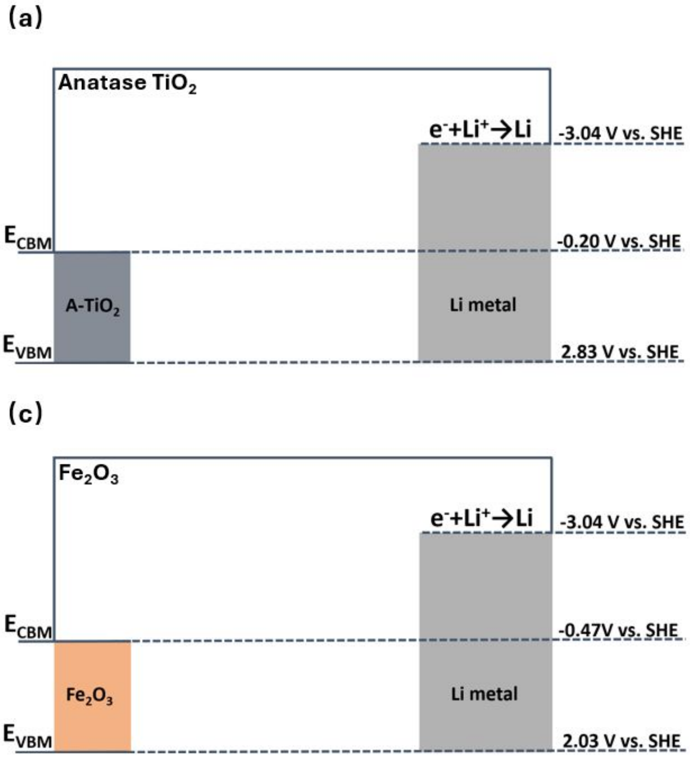

Figure S3. *Proposed band alignment against lithium plating/deplating reaction ($Li^{0/+}$) for (a) anatase TiO_2. (b) rutile TiO_2. (c) Fe_2O_3.*

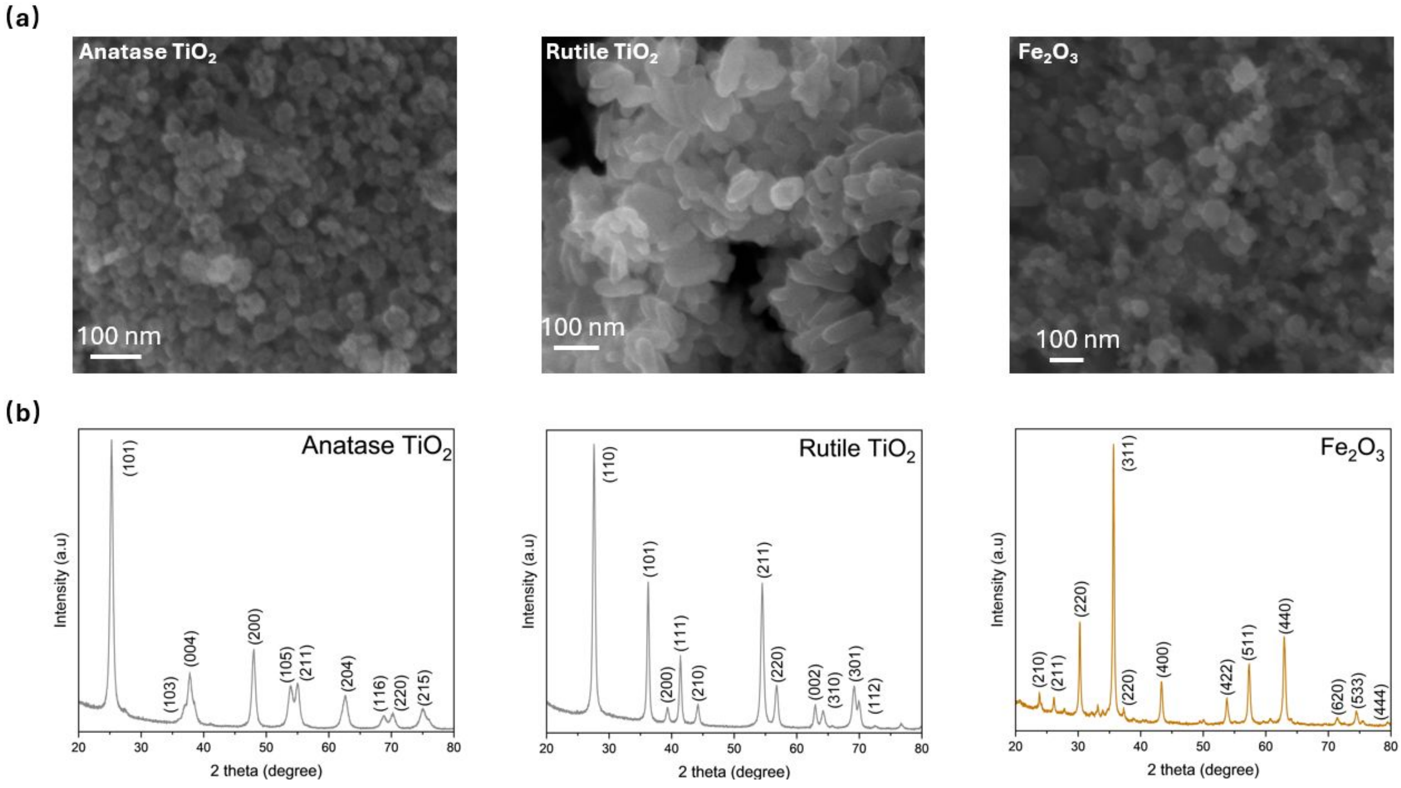

Figure S4. *(a) SEM image of anatase TiO_2, rutile TiO_2, and Fe_2O_3. (b) XRD pattern of anatase TiO_2, rutile TiO_2, and Fe_2O_3.*

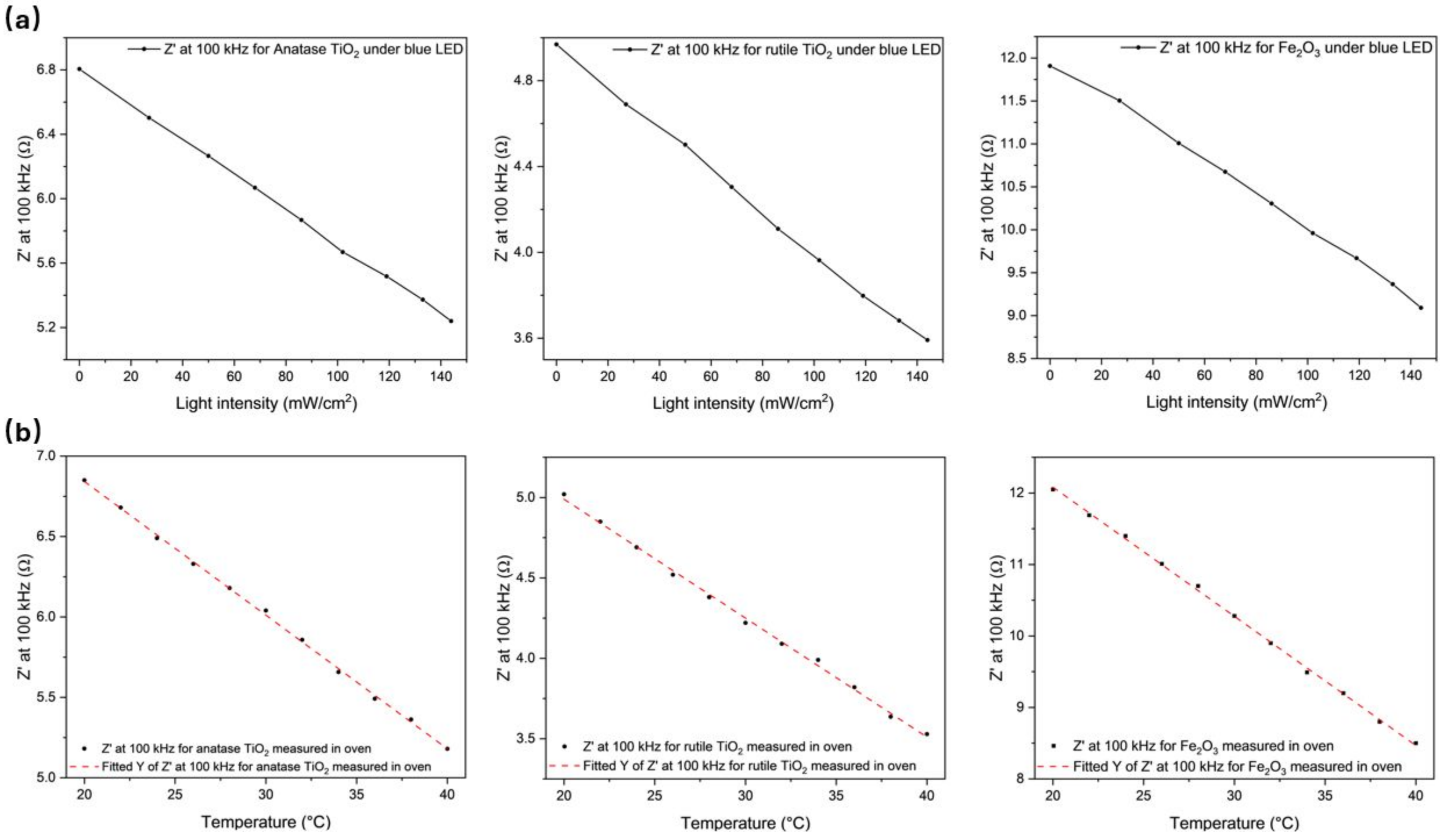

Figure S5. (a) *Impedance at 100 kHz as a function of light intensities for anatase* TiO_2*, rutile* TiO_2*, and* Fe_2O_3*.* (b) *Impedance at 100 kHz as a function of temperature for anatase* TiO_2*, rutile* TiO_2*, and* Fe_2O_3*. (Fit: Arrhenius)*

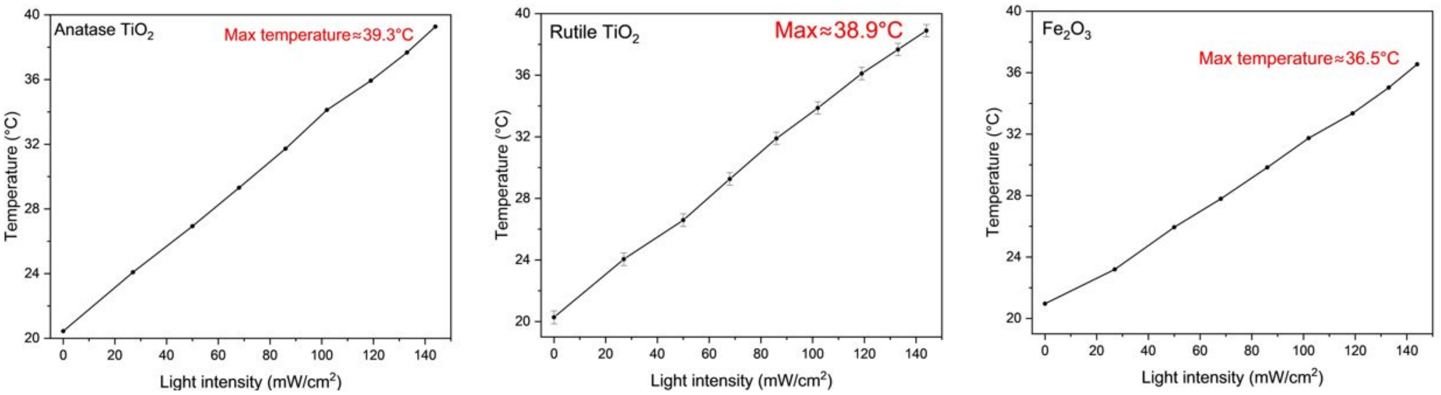

Figure S6. *Estimated internal temperature as a function of light intensity (Blue LED)* TiO_2*, rutile* TiO_2*, and* Fe_2O_3*.*

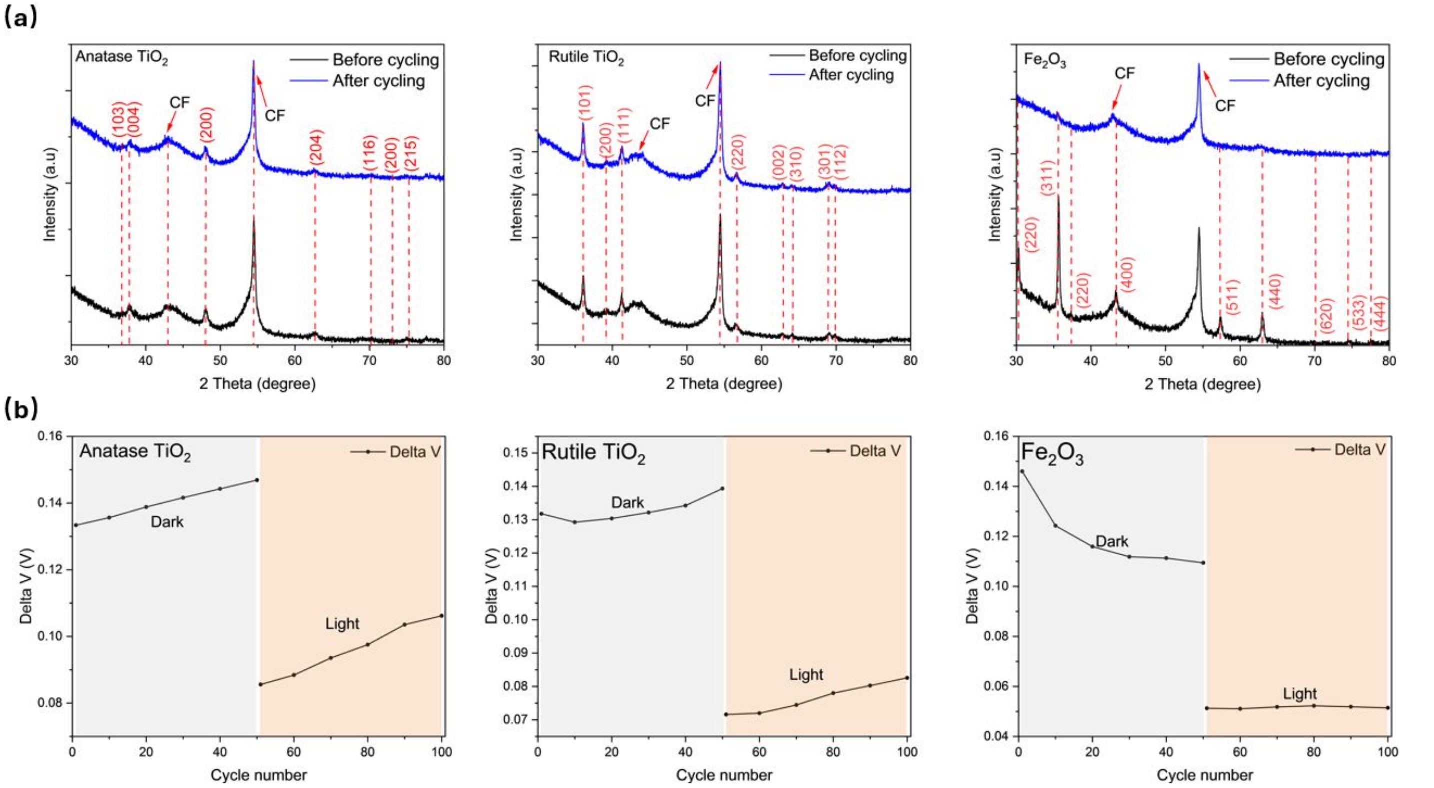

Figure S7. *(a) XRD patterns of anatase TiO_2, rutile TiO_2, and Fe_2O_3 before and after cycling. (b) The differences between nominal charge and discharge voltage (ΔV) as a function of cycle number under both dark and light conditions for anatase TiO_2, rutile TiO_2, and Fe_2O_3.*

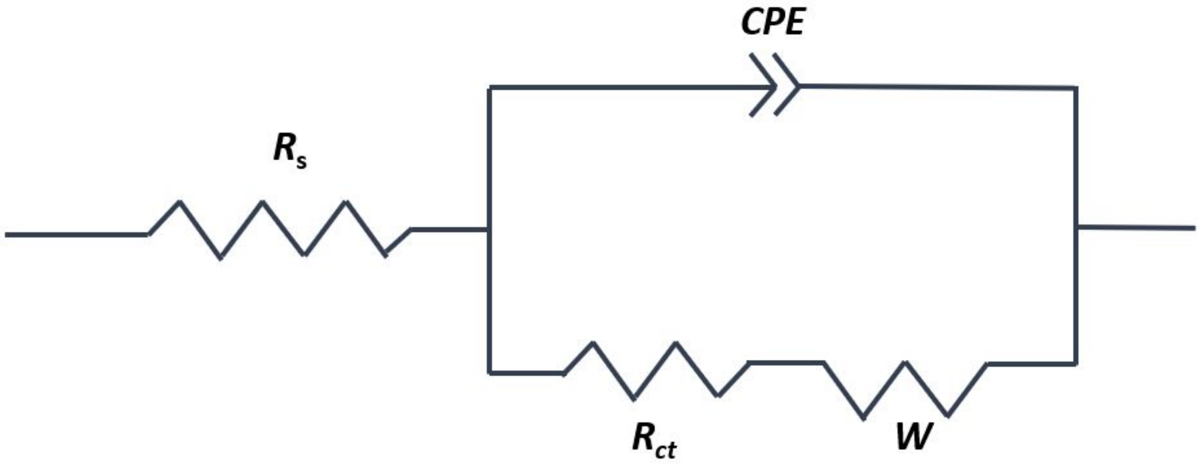

Figure S8. *Equivalent circuit for Nyquist plots for the EIS measurement, where R_s corresponds to the total resistance of the electrode, electrolyte, and separator, R_{ct} refers to the charge transfer resistance, CPE represents constant phase element corresponding to the semicircles, and W refers to the Warburg impedance.*

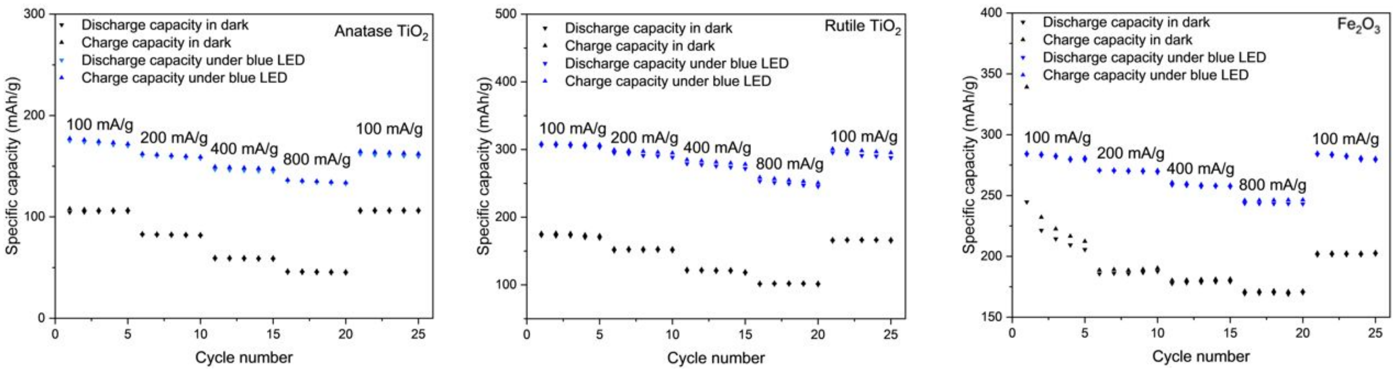

Figure S9. *Rate performance tests of the photo-LIBs in dark and illuminated conditions for anatase TiO_2, rutile TiO_2, and Fe_2O_3.*

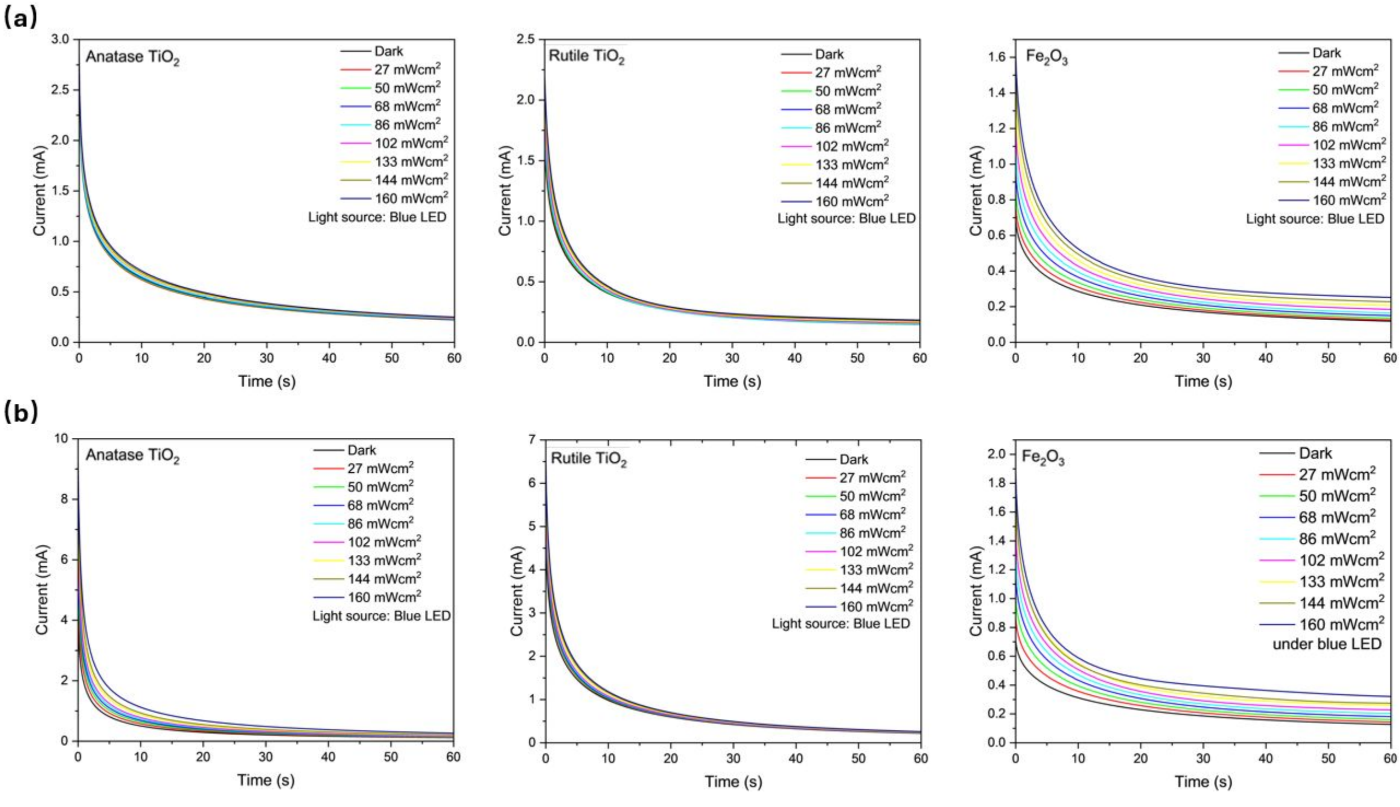

Figure S10. *Chronoamperometry curves at different light intensities (blue LED) during constant voltage hold charging at (a) 2.0 V vs. Li/Li$^+$ and (b) 3.0 V vs. Li/Li$^+$.*
